# Skin manifestations associated with systemic diseases – Part I^☆^^☆☆^

**DOI:** 10.1016/j.abd.2021.02.008

**Published:** 2021-09-17

**Authors:** Ana Luisa Sampaio, Aline Lopes Bressan, Barbara Nader Vasconcelos, Alexandre Carlos Gripp

**Affiliations:** Hospital Universitário Pedro Ernesto, Universidade do Estado do Rio de Janeiro, Rio de Janeiro, RJ, Brazil

**Keywords:** Dermatomyositis, Pyoderma gangrenosum, Sarcoidosis, Systemic lupus erythematosus, Systemic scleroderma, Sweet syndrome, Vasculitis

## Abstract

The skin demonstrates what is happening in the body in many diseases, as it reflects some internal processes on the surface. In this sense, skin as an organ, goes beyond its protective and barrier functions, as it provides clues for the identification of some systemic diseases. The dermatologist then raises diagnostic hypotheses for conditions related to all systems and refers them to the appropriate specialty. With easy access to examination by trained eyes and biopsies, the skin can present specific or non specific alterations on histopathology. In the first case this combination establishes the diagnosis of the disease itself. Non specific manifestations can occur in a variety of contexts and then histopathology is not specific of a particular disease. This article is divided into two parts that will cover large groups of diseases. In this first part, cutaneous manifestations of the main rheumatologic diseases are described, which are the ones with the greatest interface with dermatology. The authors also talk about vascular manifestations and granulomatous diseases. In the second part, endocrinological, hematological, oncological, cardiovascular, renal, gastrointestinal diseases, pruritus and its causes are discussed, and finally, the dermatological manifestations of SARS-CoV-2 coronavirus infection. The authors’ intention is that, by using direct and easily accessible language, aim to provide practical material for consultation and improvement to all dermatologists who recognize the importance of a comprehensive assessment of their patients.

## Introduction

Skin communicates with the body, often disclosing the first signs and/or symptoms of an internal disease, which may even be the only expressions of a systemic disease. In this first part, the authors will comprehensively address the cutaneous manifestations of connective tissue diseases, neutrophilic diseases, purpuras and vasculitis, and granulomatous diseases. In some of these diseases, it is possible to divide the cutaneous manifestations into specific (when biopsied, the histopathology is very helpful in the investigation of the disease) and non specific manifestations of the disease (there are no histopathological characteristics of the cases). In other diseases, this classification is not applicable. This division will be described whenever possible.

## Systemic lupus erythematosus

Lupus erythematosus (LE) is an inflammatory disease of the connective tissue with a variety of clinical presentations, characterized by the production of autoantibodies against cell constituents. The 2019 EULAR/ACR criteria for systemic lupus erythematosus (SLE) include positive ANAs at least once as a mandatory criterion, followed by additional criteria separated into seven clinical groups (constitutional, hematological, neuropsychiatric, mucocutaneous, serous, musculoskeletal, and renal) and three immunological ones (antiphospholipid antibodies, complement proteins, and SLE-specific antibodies).^1^ In the current classification, skin lesions are divided into 4 subtypes, with different scores among them according to the risk of being present in systemic lupus (non-cicatricial alopecia - 2 points; oral ulcers - 2 points, chronic discoid cutaneous OR subacute lupus – 4 points; acute cutaneous lupus – 6 points). For the diagnosis of SLE, the patient must have at least 10 points. Skin manifestations of lupus comprise a spectrum that ranges from benign and self-limited lesions to severe eruptions, which can sometimes be fatal when associated with systemic lupus erythematosus (SLE).

LE can occur as a manifestation of SLE or independently from it. Gilliam and Sontheimer classified cutaneous LE into three forms, based strictly on the clinical appearance of the cutaneous lesions, comprising three forms: chronic cutaneous LE, subacute cutaneous, and acute cutaneous LE. Gilliam, however, expanded this classification based on specific and non-specific clinical and histopathological characteristics.^2^ LE patients may have more than one clinical form of the disease. Non specific manifestations are those that do not have histopathological characteristics of lupus erythematosus but provide “clues” regarding the diagnosis and prognosis. In contrast, specific manifestations of LE encompass the subtypes of cutaneous lupus erythematosus (CLE), which are chronic cutaneous lupus erythematosus (CCLE), subacute (SACLE), and acute (ACLE). The cutaneous manifestations of SLE are described in Table 1.

**Table 1:** Specific and non specific lesions of lupus erythematosus.

Specific manifestations of LE	Non specific manifestations of LE
1. Acute Cutaneous Lupus Erythematosus (ACLE)	1. Vasculopathies
Localized (malar erythema)	Vasculitis
Photosensitivity	Urticarial vasculitis
Generalized	Livedo reticularis/Livedo racemosa
2. Subacute cutaneous lupus erythematosus (SACLE)	Livedoid vasculopathy
Annular	Degos' disease like
Papulosquamous	Raynaud’s phenomnon
3. Chronic cutaneous lupus erythematosus (CCLE)	Erythromelalgia
Discoid LE (localized/generalized)	Gangrene of the extremities
Hypertrophic/verrucous LE	Pyoderma gangrenosum lesion
Lupus panniculitis/Lupus profundus	2. Non specific bullous lesions
Mucosal lupus	Bullous lupus
Tumid LE	3. Non-specific alopecia:
Chilblain lupus	Telogen effluvium (disease activity)
Comedonic lupus	“Lupus hair”
4. Atypical specific LE lesions	4. Calcinosis
Small papules on the neck	5. Pigmentary changes
Lupus on the elbow	6. Nail changes
5. Specific bullous lesions:	7. Mucosal lesions
Lupus “with bullous lesions” (ACLE, SACLE, DLE)	Aphthous lesions
6. Alopecia with specific lesions:	8. Multiple dermatofibromas
ACLE	9. Rheumatoid nodules
SACLE	10. Occasional manifestations:
DLE	Urticaria/Angioedema
7. Specific lesions in mucous membranes:	Lichen planus
ACLE	Neutrophilic dermatoses
CCLE	Erythema multiforme
	Seborrheic dermatitis
	Vitiligo

## LE specific lesions

### Acute cutaneous lupus erythematosus (ACLE)

**Figure 1:**
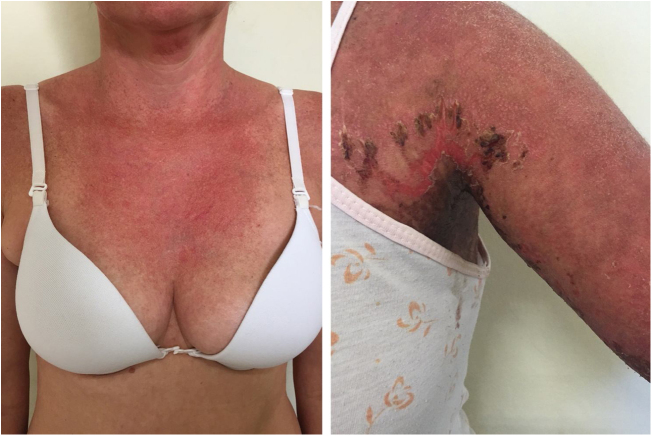
Acute LE and NET-like. Left: acute LE: diffuse erythema on the neck, in a sun-exposed area. Right: erythema and diffuse edema with exulcerations and crust formation, characterizing the more evident detachment of the skin in the axillary region (“lupus with bullae”).

Localized ACLE: facial erythema commonly called “malar rash” is characterized by malar and nasal erythema and edema that spare the nasolabial fold. The erythema is induced by ultraviolet light, can last hours, days, weeks, spontaneously regress and recur. In cases of intense photo exposure, the skin surface may show necrosis, the so-called “lupus with bullae” (entity distinct from bullous lupus) and, if generalized, it resembles toxic epidermal necrolysis (TEN), being called TEN-like lupus (Fig. 1). High phototypes may present clinically with hyperpigmentation or hypopigmentation, even after resolution of inflammation. It is commonly associated with SLE with positive anti-Ro (SSA) antibodies. Photosensitivity is an important factor in LE in all skin types. UVB and UVA radiation can aggravate skin disease.

Generalized ACLE: maculopapular erythema involving sun-exposed areas (hands and arms) in the interarticular regions. Skin over the finger joints is usually spared, as opposed to Gottron’s sign in dermatomyositis, in which the erythema is juxta-articular.^3^

Histopathology: vacuolar interface dermatitis, apoptotic keratinocytes, lymphohistiocytic infiltrate in the superficial dermis and mucin deposition in the dermis.^4^

### Subacute cutaneous lupus erythematosus (SACLE)

**Figure 2:**
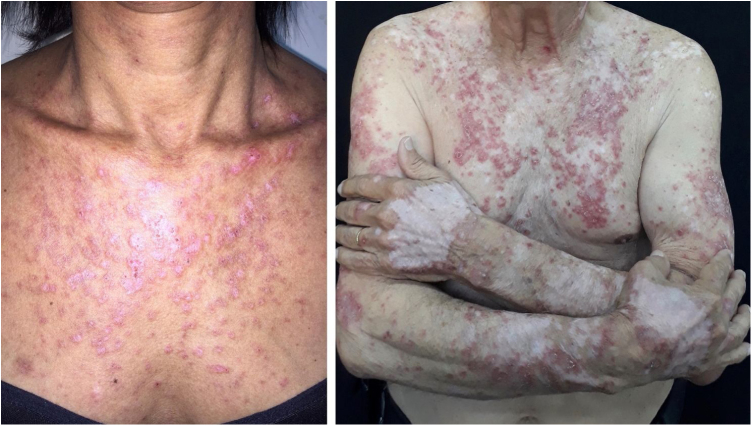
Subacute LE. Subacute lupus erythematosus: erythematous squamous lesions and vitiligous-like lesions affecting the anterior trunk and extensor surface of the upper limbs and dorsum of the hands.

It initially manifests with small erythematous and slightly scaling papules that may evolve into psoriasiform plaques (papulosquamous form) or annular erythematous plaques with central clearing and peripheral scaling (annular form). The two types of lesions may have a superimposed appearance and show vesicles and crusts or even hemorrhagic bullae at the periphery, due to damage to the epidermis and basal layer by photo exposure – also called “lupus with bullae”.^3^ Sun-exposed areas are most commonly affected, such as the extensor surface of the upper limbs, neck, and upper torso (known as the “V-neck area”). When they regress, the lesions leave residual telangiectasias, hyper or hypochromia. Pigment loss can be so intense as to show vitiligo characteristics (Fig. 2). Scarring and atrophy are not seen. SACLE seems to be associated with SLE in approximately 50% of cases and on histopathology shows basal layer vacuolization, perivascular lymphocytic infiltrate, mucin deposition in the dermis, and, when compared to CCLE, there is less hyperkeratosis and follicular horn plugs.^4^

### Chronic cutaneous lupus erythematosus (CCLE)

Classic discoid LE: these are CCLE lesions characterized by erythema, telangiectasias, adherent lamellar scales, with a discoid appearance, which vary from thin to thick, hypo, hyper, or achromic plaques that, when not effectively treated, leave atrophy and scars. In general, they are asymptomatic, single or multiple, preferentially located in sun-exposed areas, especially on the face and auricle. The more extensive and below the neck location (disseminated discoid CCLE), the greater the risk of developing SLE. It is estimated that 5% to 30% of patients with CCLE develop the systemic disease. Acneiform lesions, including comedones and punctate scars, are atypical presentations called comedonic lupus.^5^ Scalp involvement occurs in approximately 60% of patients with DLE, and it may be the only affected region in approximately 10% of cases. Cicatricial alopecia occurs secondary to follicular destruction if there is no timely adequate treatment.

Hypertrophic or verrucous LE: a rare variant of CCLE, characterized by verrucous plaques in which scaling is replaced by hyperkeratosis. Lesions are preferably located on the extensor surface of the limbs, but also on the back and facial regions. These lesions are usually pruriginous. There may be overlapping with lichen planus (LP), called LE/LP. There are few systemic symptoms and laboratory alterations. The lesions are difficult to treat with conventional therapy but may respond to oral retinoids.^6^

Lupus panniculitis: it clinically manifests with subcutaneous stony nodules that, if not adequately treated, lead to subcutaneous atrophy and calcification, and occasional ulceration. It occurs in the malar and temporal regions, upper limbs, thighs, buttocks, and breasts. Histopathology shows a lobular panniculitis with lymphocytic infiltration, which can eventually organize into lymphoid follicles with germinative centers and reach the septa. There may be a lymphocytic vasculitis and an infiltrate containing eosinophils. The term ‘lupus profundus’ (of Kaposi-Irgang type) is often used as a synonym for lupus panniculitis; however, some authors reserve this term for lupus panniculitis with discoid lupus lesions on the skin surface.

Tumid lupus: it is characterized by erythematous-violaceous infiltrated plaques or papules, without scaling, which mainly affects sun-exposed areas, such as the face and neck. Lesions tend to regress without scarring or atrophy. Patients with tumid LE rarely meet the criteria for the systemic form of the disease.

Chilblain lupus: a rare variety of CCLE, more common in cold climates, located in the extremities or tip of the nose. Histopathology shows hyperkeratosis, follicular horn plugs, liquefaction degeneration of the basal layer with thickening of the basement membrane, lymphocytic infiltrate near the dermo-epidermal junction, and presence of mucin in the dermis.^7^

### Atypical specific LE lesions

Papular lupus (neck): small erythematous papular lesions found especially on the neck area of young women. It is clinically distinct from LE-associated cutaneous mucinosis.^7^

Lupus on the elbow: it consists of erythematous papules or plaques on the elbows, usually bilateral and recurrent, which have specific histopathology for LE. This unique location seems to be associated with an increased risk for systemic involvement.^8^

### Non-specific LE lesions

Vasculitis: leukocytoclastic vasculitis is the most frequent non specific manifestation of SLE (20%–40%). Lesions occur, preferably, on the extremities of the lower limbs or in areas of pressure or trauma and may present as small-vessel cutaneous vasculitis, with macules, papules, or palpable purpuric lesions. They are related to disease activity.

Urticarial vasculitis: Urticarial lesions that last more than 24 hours, accompanied by a burning or stinging sensation and which can leave hyperpigmentation when regressing. Hypocomplementemia indicates worse prognosis (greater probability of renal involvement).

Vasculopathies:

a) Livedo reticularis or livedo racemosa: it is usually found in patients with antiphospholipid syndrome (APS). However, many of these patients also have LE associated with positive anticardiolipin antibody and/or lupus anticoagulant;
b) Livedoid vasculopathy and *Atrophie blanche* (white atrophy): it is considered a localized thrombotic vasculopathy that, after healing, leaves an area of ​​white atrophy, which is usually painful;
c) Degos’ disease-like: small erythematous papular lesions (2 to 5 mm) with a central depression, which regress leaving white atrophic scars with telangiectasias at the periphery. It differs from true Degos’ disease because it is associated with LE and a more benign and self-limited course;
d) Raynaud’s phenomenon: It is observed in 10%–44% (~33%) of LE cases. It is associated with the presence of Anti-U_1_RNP antibody (60%–90% of cases);
e) Erythromelalgia: erythema, heat, intolerable pain predominantly in hands and feet. It worsens with exposure to heat and is relieved with exposure to cold;
f) Gangrene of the extremities: it is a thrombotic phenomenon. Antiphospholipid antibody, cryoglobulinemia, and infective endocarditis must be investigated.

Bullous lupus: the histopathology of bullous lupus is similar to that of dermatitis herpetiformis or epidermolysis bullosa acquisita. The bullae have an acute onset, predilection for the trunk, cervical region, and proximal extremities. There are five diagnostic criteria proposed by the American College of Rheumatology (ACR), namely: 1) Non-cicatricial acquired bullous eruptions, appearing in a photo exposed area, but not limited to it; 2) Histopathology showing a subepidermal bullous lesion, with a predominantly neutrophilic infiltrate in the dermis and basement membrane area; 3) Direct immunofluorescence showing deposits of IgG, IgA, IgM, and C3 on the basement membrane zone of perilesional skin; 4) Circulating antibodies against type VII collagen confirmed by indirect immunofluorescence using salt-split skin, immunoblotting or immunoprecipitation techniques; 5) Immunoglobulin deposits in anchoring fibrils and type VII collagen on immunoelectron microscopy.^6^, ^7^, ^8^, ^9^, ^10^, ^11^

Alopecia:

a) Specific: ACLE and SACLE lesions on the scalp can generate non-cicatricial alopecia. On the other hand, the CCLE lesion, if not treated in a timely manner, can evolve into a cicatricial lesion;
b) Non specific: telogen effluvium and anagen effluvium generally indicate disease activity (anagen can occur quickly after the onset of major SLE reactivation or after treatment with cyclophosphamide or methotrexate). Lupus hair: short, dry-looking hair shafts in the frontal implantation line. It affects women with long-term SLE.

Papulonodular mucinosis: it indicates disease activity and presents a strong association with renal involvement. It is caused by mucin deposition in the dermis, in an area with or without LE changes, located on the upper trunk and extremities.

Calcinosis: hard and irregular nodules in the subcutaneous tissue that can be eliminated to the surface, with white to yellowish material discharge.

Pigmentary changes: hypo and hyperpigmentation are common. Attention should be paid to hyperpigmentation caused by antimalarials: grayish beard, hair and eyelashes, diffuse hyperpigmentationand of the nail edges.

Nail changes: hemorrhagic splinters, pitting, leukonychia, onycholysis (more common), digital clubbing and red lunula, telangiectasias in the cuticles, and dyschromia.

Mucosal lesions: any mucosa can be affected in LE. Occasionally, the lesions may present with specific histopathology (of CCLE or ACLE); aphthous ulcerations and erosions are observed in 5% to 10% of patients with SLE.

Eruptive dermatofibromas: the appearance of multiple dermatofibromas may accompany SLE and other collagen diseases such as Sjögren’s syndrome.

Rheumatoid nodules: they are present in 5% to 10% of patients with SLE, and mainly affect the hands and elbows.

Occasional manifestations: urticaria/angioedema, lichen planus, erythema elevatum diutinum, pyoderma gangrenosum, Sweet’s syndrome, amicrobial pustulosis of the folds, polymorphic erythema, extensive and refractory seborrheic dermatitis, vitiligo, factitious dermatitis, and exanthema.

## Dermatomyositis

Dermatomyositis (DM) is an idiopathic, clinically heterogeneous inflammatory disease that can be difficult to diagnose. It encompasses various cutaneous manifestations that may or may not parallel myositis and systemic involvement over time and/or disease severity.

Bohan and Peter, in 1975, suggested the use of five criteria to diagnose dermatomyositis. These include: 1) Symmetrical proximal muscle weakness that progresses over a period of weeks to months; 2) Elevated serum levels of muscle enzymes; 3) Abnormal electroneuromyography; 4) Abnormal muscle biopsy; 5) The presence of cutaneous manifestations typical of dermatomyositis. New criteria, by Dalakas and Hohlfeld, in 2003, highlighted the importance of histopathological evaluation of the muscle, improving its specificity, albeit becoming less sensitive. These are useful criteria for evaluation but are unnecessary in patients with characteristic skin disease, particularly those with proximal muscle weakness and elevated muscle enzymes.

**Figure 3:**
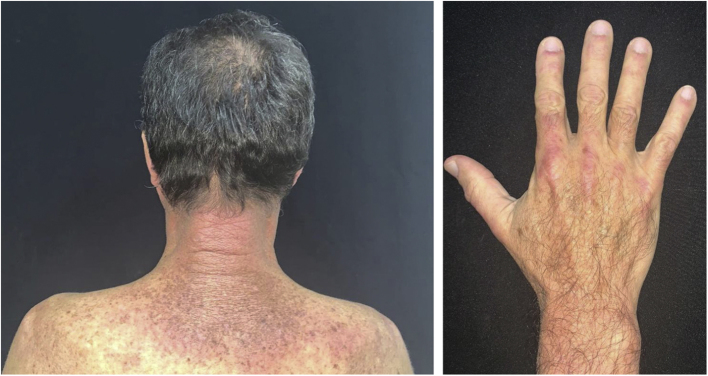
Dermatomyositis. Left: characteristic erythema on the posterior cervical region and upper back (“shawl sign”). Right: erythema on the back of the hands (Gottron’s sign).

The clinical course of dermatomyositis skin lesions is not necessarily parallel to that of muscle disease and may precede or follow myositis. In more than half of patients, cutaneous manifestations precede muscle involvement by months or years. Moreover, muscle disease activity is not reflected by skin disease activity. Cutaneous involvement can be divided into seven types: pathognomonic, characteristic, compatible, less common, rare, recently described, and non-specific cutaneous manifestations.^9^ Pathognomonic cutaneous manifestations (heliotrope and Gottron’s papules) and the others will be reported below (Fig. 3).

Gottron’s papules: they consist of erythematous violaceous papules covering the interphalangeal and metacarpophalangeal joints.

Heliotrope: it consists of pink to violaceous erythema, with or without edema, involving the periorbital skin. The upper eyelid is the most affected site.

Gottron’s sign: it consists of erythematous-violaceous macules over other joints, such as elbows or knees.

Photosensitivity: the clinical picture is characterized by intense photosensitivity, with erythematous-violaceous lesions on the scalp, neck, shoulders, extensor surfaces of the upper extremities, upper chest region (“V-neck sign”), and upper back region (“shawl sign”). The malar erythema reaches the nasolabial fold as opposed to malar erythema in lupus erythematosus, which spares it.

Pruritus: it can be intense and persistent, affecting the patient’s quality of life. Moreover, its presence may be useful to clinically differentiate dermatomyositis from lupus erythematosus, where there is no marked pruritus. It is often resistant to treatment with topical antihistamines and corticosteroids. Erythematous-squamous lesions may also be present on the lateral aspect of the thighs (“holster sign”).

Scalp lesions: scalp involvement is characterized by atrophic, erythematous, and squamous plaques. There is marked pruritus in this region, more than can be observed from the clinical findings. These manifestations of dermatomyositis can be misdiagnosed, especially in the initial phase, as seborrheic dermatitis or psoriasis. Mild to moderate non-cicatricial alopecia may occur.

Nail fold lesions: periungual telangiectasia (apparent or detected by digital microscopy), cuticular hypertrophy and dystrophy, and small hemorrhagic infarcts are typical alterations of the nail folds.

Vasculitis: it can manifest as palpable purpura, urticaria-like lesions, livedo reticularis, nail fold infarction, digital or oral ulceration; less commonly as vesico-bullous, erosive, necrotic, and ulcerative lesions. Cutaneous vasculitis occurs mainly in juvenile dermatomyositis, and usually as leukocytoclastic vasculitis. The presence of vesico-bullous and erosive skin lesions or cutaneous vasculitis should lead to a diagnostic investigation for possible underlying malignancy.

Cutaneous calcinosis: there are calcified deposits in the skin and subcutaneous tissue. According to serum calcium/phosphate levels, cutaneous calcinosis can be classified into four subtypes: dystrophic, metastatic, iatrogenic, and idiopathic. Dystrophic calcinosis occurs in damaged tissues and is associated with several connective tissue diseases, especially dermatomyositis, with serum calcium/phosphate levels within normal limits. Its pathophysiology remains unclear. Calcinosis is more common in juvenile dermatomyositis (30%–70% of cases) than in adults (10% of cases). It presents as superficial or subcutaneous nodules, found mainly in sites of repeated trauma, such as the gluteal region, elbows, and knees. Calcinosis located on the extensor surface of the extremities can ulcerate, leading to chronic, difficult-to-heal ulcers.

“Mechanic’s hands”: characterized by hyperkeratosis, scaling, and fissures in the fingers or even the palms of the hands. The lesions are distributed along the ulnar surface of the thumb and radial aspect of the fingers and are more prominent on the index and middle fingers, with rare extension to the palm. It is characteristic of the anti-synthetase syndrome, which also includes the findings of arthritis, interstitial lung disease, and Raynaud’s phenomenon. Mechanic’s hands can also be seen outside the anti-synthetase scenario, in cases of polymyositis, classic and amyopathic dermatomyositis.

Wong Variant: it is characterized by hyperkeratotic, follicular erythematous papules on the extensor side of the extremities, sometimes associated with palmar keratoderma.

Flagellate erythema: it is characterized by erythematous, edematous, linear, pruritic and/or painful lesions (as if produced by whipping) located on the trunk. It can occur in association with dermatomyositis, adult-onset Still’s disease, treatment with systemic bleomycin, shiitake consumption, and exposure to jellyfish.

Panniculitis: It occurs in the juvenile and adult forms, as indurated, isolated, or confluent nodules on the gluteal region, upper limbs, thighs, and abdomen. Some lesions are followed by calcification. Histopathological examination shows lobular panniculitis with lymphoplasmacytic infiltrate, liquefaction at the dermo-epidermal junction, and membranocystic changes. The pathogenesis of panniculitis in dermatomyositis is unclear.

Vesiculobullous eruptions: vesicles or bullae may rarely develop on the dorsal surfaces of the hands or forearms, and in other areas. These eruptions can be caused by photosensitivity, with histopathological findings of liquefaction degeneration of the basal layer of the epidermis, subepidermal edema, mucin deposition in the upper dermis, or complications from autoimmune bullous diseases.

Erythroderma: it is rare and is associated with malignancy.

Oral mucosal lesions: oral mucosal involvement includes telangiectasias, edema, erosions, ulcers, and leukoplakia-like areas. Gingival telangiectasia probably represents a diagnostic finding analogous to nail fold telangiectasia. Hyposalivation is common and may explain an increased prevalence of dental caries in these patients.

Inverse Gottron’s papules: a very rare manifestation of dermatomyositis. Located on the palmar surface of the interphalangeal joints (unlike classic Gottron’s papules). They present as localized white triangular hyperkeratosis.

Recently described cutaneous manifestations: these are rare and include inverse Gottron’s papules, digital ulcerations, Gottron’s papules (or sign) with ulceration, and “hiker’s feet” (mechanic’s feet).

Raynaud’s phenomenon: mainly seen in patients with dermatomyositis and overlapping autoimmune diseases or in the anti-synthetase syndrome. It is a non-specific manifestation of the disease.^9^

A skin biopsy can help to differentiate dermatomyositis from other papulosquamous or eczematous diseases but cannot be used to differentiate dermatomyositis from lupus erythematosus. Classically, the skin biopsy in dermatomyositis shows hyperkeratosis, epidermal thinning, vacuolar interface dermatitis, thickening of the epithelial basement membrane, dermal edema, pigment incontinence, mucin deposits, and a perivascular infiltrate consisting of CD4+ lymphocytes. These findings are observed in the pathognomonic lesions that are characteristic of dermatomyositis; however, they are not seen in mechanic’s hands, panniculitis, cutaneous vasculitis, urticaria, flagellate erythema, and follicular hyperkeratosis.

Dermatomyositis is a heterogeneous disorder with multiple phenotypes, including myositis, dermatitis, and interstitial lung disease. Recently identified myositis-specific autoantibodies have been associated with distinct clinical features. These autoantibodies are highly specific for the disease. For example, melanoma differentiation-associated protein 5 (MDA-5) antibodies have high specificity for clinically amyopathic DM with rapidly progressive lung disease. Anti-transcriptional intermediary factor 1-γ antibody, found in juvenile and adult DM patients, is closely related to neoplasms, especially in elderly patients. Patients with anti-aminoacyl-transfer RNA synthetase (ARS) antibodies share characteristic clinical symptoms, including myositis, pulmonary disease, arthritis/arthralgia, Raynaud’s phenomenon, and fever; therefore, the term “anti-synthetase syndrome” is also used (Table 2).^10^

**Table 2:** Autoantigens and dermatomyositis phenotypes.

Autoantigen	Clinical manifestation
MDA5	Amyopathic DM; interstitial lung disease
TIF1	Juvenile DM; DM associated with neoplasm
Mi2	Classic DM
ARS	Anti-synthetase syndrome; chronic interstitial lung disease
NXP2	Juvenile and adult DM
SAE	Amyopathic DM; severe myositis

## Rheumatoid arthritis

Rheumatoid arthritis (RA) is an autoimmune disease characterized by inflammation and the development of joint deformities and by association with the rheumatoid factor (75% of patients with RA and in 5% to 10% of healthy individuals) and with the second-generation anti-cyclic citrullinated peptide (CCP) antibody (specificity from 90% to 95%).^11^

The lesions do not have typical RA histopathology but are suggestive; some are specific to the disease and others have a greater association. They include neutrophilic lesions – pyoderma gangrenosum and rheumatoid neutrophilic dermatosis; palisaded granuloma – rheumatoid nodule, palisaded granulomatous and neutrophilic dermatitis, granulomatous interstitial dermatitis; vascular lesions – small and medium-vessel cutaneous vasculitis, Bywater’s lesions, erythema elevatum diutinum, petechiae and palmar and subungual erythema; nail changes – onychorrhexis, onychomadesis, onycholysis, digital clubbing, red lunula, and inverse nail pterygium; other changes – amyloidosis and chronic spontaneous urticaria.^11^

RA-related subcutaneous nodules include classic rheumatoid nodules, accelerated rheumatoid nodulosis, and rheumatoid nodulosis. ^11^

Classic rheumatoid nodules: indurated single or multiple subcutaneous nodular lesions of variable size, preferably located on the extensor surfaces of the forearms and elbows. They occurs in 20% to 30% of cases and 90% are associated with the presence of the rheumatoid factor at high titers but unrelated to disease severity or progression.^11^ Smokers are at greater risk of developing them. They can also be seen over finger joints, sacral region, ischial tuberosity, Achilles tendon, pre-auricular region, and scalp. When large, they can cause nerve compression, ulcerate, and be cosmetically unpleasant. Histopathologically, they exhibit large nodules in the deep dermis and hypodermis, consisting of fibrin and surrounded by palisaded granulomas.

Accelerated rheumatoid nodulosis: it is characterized by previous subcutaneous nodules (preferably over hand joints) that grow suddenly and rapidly and are independent of joint disease activity.^11^ They can be associated with the treatment of RA, especially with methotrexate.

Rheumatoid nodulosis: multiple subcutaneous rheumatoid nodules without the presence of severe RA and without systemic manifestations, more common in middle-aged men (30 to 50 years), with a self-limited and benign course. There are also reports in children, not associated with the development of RA, and lesions in atypical areas such as the scalp, pretibial region, knees, feet, and perimalleolar region.

The vascular manifestations of RA are not specific and their prototype is rheumatoid vasculitis, which consists of small and medium-vessel leukocytoclastic vasculitis, due to deposits of immune complexes that affect both the skin and the mesenteric vessels, central nervous system, and heart, in 2% to 5% of patients with long-term, erosive disease with high titers of rheumatoid factor. Depending on the caliber and location of the affected vessel, it may manifest as a purpuric macular or papular lesion, retiform purpura, livedo reticularis, subcutaneous nodules, and ulcers (usually perimalleolar ones). Digital infarcts secondary to Raynaud’s phenomenon can also be observed. Small-vessel cutaneous vasculitis can also occur as a reactive phenomenon in the presence of disease activity. What differentiates cutaneous vasculitis from rheumatoid vasculitis is that the latter usually is sudden and extensive, with simultaneous involvement of internal organs and a high mortality rate, whereas the first follows a more benign course. Peripheral neuropathy in the context of rheumatoid vasculitis indicates a worse prognosis and must be quickly treated.

Granulomatous and neutrophilic lesions have also been reported, such as neutrophilic rheumatoid dermatitis, granulomatous and neutrophilic palisaded dermatitis, granulomatous interstitial dermatitis with arthritis, pyoderma gangrenosum, and Sweet’s syndrome. Within the group of granulomatous lesions, neutrophilic granulomatous dermatitis is a suspicious manifestation of RA, albeit rare. It manifests as urticarial symmetrical erythematous papules, plaques, nodules, or bullae in women (2:1), with erosive disease and high RF titers, on the extensor surfaces of the joints (mainly dorsum of hands and forearms). Histopathology discloses a neutrophilic, leukocytoclasia, and endothelial edema without fibrinoid necrosis (i.e., no evidence of vasculitis). Severe spongiosis can cause clinical vesicle/bullous lesions. Its course follows that of RA activity.

There is debate whether the other granulomatous lesions are within the spectrum of palisaded granulomatous dermatitis, but with some clinical and histopathological differences. They can also be called granulomatous interstitial dermatitis with arthritis, Churg-Strauss granuloma, rheumatoid papules, ulcerating rheumatoid necrobiosis, and extravascular necrotizing granuloma of Winkelmann. Palisaded granulomatous dermatitis presents with painful normochromic papules and nodules on the extensor surfaces of the limbs, with umbilication or central crust. Urticarial and annular lesions have been described, in addition to livedo reticularis.

Interstitial granulomatous dermatitis with arthritis presents as annular papules, erythematous-violaceous nodules and plaques, and subcutaneous linear cords, symmetrical on the lateral side of the trunk and also seen on the inner thighs, in middle-aged women with high RF. It is not exclusive to RA, as it can occur in other collagenoses. It may also be associated with an underlying lymphoproliferative malignant neoplasm and HIV infection. Drug-induced granulomatous dermatitis (lipid-lowering agents, anticonvulsant, antihistamines, and anti-TNF drugs)^11^ may be clinically similar but presents with vacuolar interface dermatitis. Histopathology basically shows a histiocytic infiltrate with a palisaded border, a center with collagen degeneration, and a neutrophilic infiltrate. In palisaded granulomatous dermatitis, in addition to the abovementioned histopathological findings, there may be leukocytoclastic vasculitis.

Felty’s syndrome: This is a rare, extra-articular manifestation of seropositive RA, characterized by a triad comprising arthritis, neutropenia, and splenomegaly. The accompanying skin lesions are rheumatoid nodules (as it is a long-term erosive disease with high RF titles) and leg ulcers. It is associated with a poor prognosis, with a mortality rate of 25% (usually from sepsis).

## Still’s disease

Still’s disease (or juvenile idiopathic arthritis, JIA) is a rheumatologic disease that courses with childhood or adolescent-onset arthritis. It is currently classified into 7 subtypes.^12^ The systemic subtype of JIA is the one that courses with classic dermatological lesions, consisting of a macular and papular, morbilliform or evanescent urticarial eruption of erythematous to orange color (salmon), which occurs during episodes of fever (usually in the evening) and may precede or accompany arthritis. Histopathology is non specific and shows a sparse lymphocytic and histiocytic infiltrate in the superficial and perivascular dermis.

**Figure 4:**
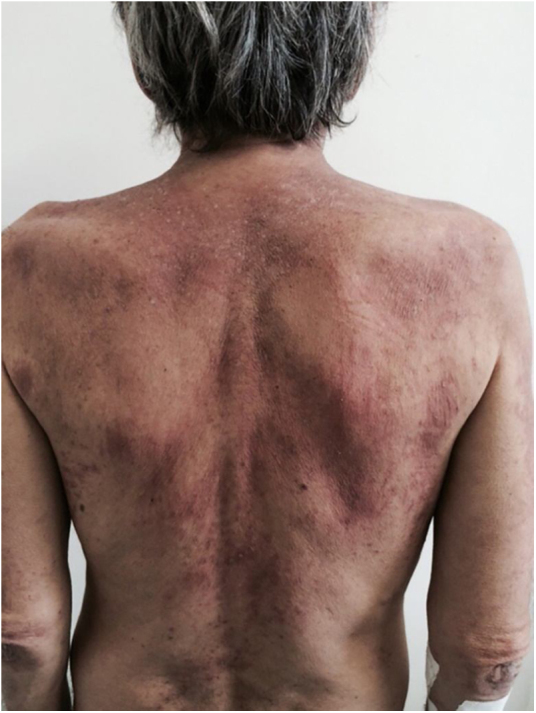
Adult-onset Still’s disease – flagellated lesions. Linear flagellate macules of adult-onset Still’s disease.

There is a type of adult-onset Still's disease that is usually seen in women before 30 years of age, in which the evanescent lesions described above occur, as well as atypical lesions of different clinical presentations such as urticarial papules, lichenoid papules, lesions similar to dermographism (Fig. 4), similar to dermatomyositis, prurigo pigmentosa, and lichen amyloidosus. All these manifestations have the same histopathology (parakeratotic foci, single or multiple necrotic keratinocytes in the Malpighian layer, and a neutrophilic infiltrate in the upper perivascular dermis. Absence of eosinophils). These “atypical” persistent lesions have an estimated frequency of 29% to 78% in patients with adult-onset Still’s disease and, when present, they indicate worse prognosis. They are therefore markers of disease severity.^13^ Other cutaneous manifestations associated with Still's disease that have been described include alopecia, cutaneous pain, acneiform lesions, pruritus, non-caseous granulomas, eczema-like lesions, urticaria, angioedema, palmoplantar vesicopustules, dichromic erythema migrans, generalized *peau d’orange*-like infiltration equal to diffuse cutaneous mucinosis, and persistent generalized erythema.^14^, ^15^

## Raynaud’s phenomenon

Raynaud’s phenomenon is defined by the change in the color of the fingers that corresponds to vasospasm (pallor and cyanosis), followed or not by vasodilation (erythema), due to sudden exposure to low temperatures on days with mild temperatures. It can be classified as primary (with milder symptoms) or secondary to an underlying disease, such as a connective tissue disease. In this case, the disease is more aggressive, with a greater risk of ulcerations and necrosis on acral extremities. It usually affects the fingers, but it has been described on the ears and nipples.

In 2014, Maverakis et al. established that the diagnosis is made by meeting three of the criteria below: 1) presence of clinical findings suggestive of Raynaud (pallor - cyanosis - erythema); 2) normal capillaroscopy; 3) negative physical examination for secondary causes (ulcerations, gangrene, sclerodactyly, calcinosis, cutaneous fibrosis; 4) no previous history of connective tissue disease; 5) negative or low ANA titers (1:40).

In a patient with Raynaud’s phenomenon, capillaroscopy must be performed, in addition to a complete investigation including a thorough history and physical examination and an autoantibody panel. Capillaroscopy changes in a patient with Raynaud’s phenomenon are a risk marker for the disease to be secondary to a connective tissue disease.

### Causes associated with secondary Raynaud’s phenomenon

Autoimmune diseases: scleroderma, lupus erythematosus, Sjögren’s syndrome, dermatomyositis, mixed connective tissue disease, undifferentiated connective tissue disease, overlap syndrome.

Vascular diseases: atherosclerosis, thoracic outlet syndrome, vasculitis.

Hematological/oncological: POEMS syndrome, paraproteinemias, cryoglobulinemias, paraneoplastic syndromes.

Drugs: sympathomimetics, bleomycin, interferon, ergotamine, nicotine, polyvinyl chloride (PVC).

Endocrinological: hypothyroidism, hyperthyroidism, autoimmune thyroiditis.^16^

## Sjogren’s syndrome

It is an autoimmune disease of unknown etiology and can be classified as primary (pSS) or secondary (sSS) if associated with another connective tissue disease. The cutaneous manifestations of pSS are underestimated so that dermatologists often do not make this diagnosis. There are no specific cutaneous manifestations of SS.^17^ That is why it is difficult to attain a conclusive diagnosis of pSS, as the disease shares non-specific clinical and immunological characteristics with other connective tissue diseases.^18^ The cutaneous manifestations are: 1) sicca syndrome: xerostomia, xerophthalmia, and xerosis; 2) Raynaud’s phenomenon; 3) cutaneous vasculitis: small-vessel (IgG/IgM), cryoglobulinemia and erythema elevatum diutinum (rare); 4) positive antiRo (SSA) annular lesions; 5) localized nodular cutaneous amyloidosis; 6) Pruritus; 7) photosensitivity; 8) associated dermatoses: alopecia (areata), vitiligo, lichen planus, anetoderma, Sweet’s syndrome, granulomatous panniculitis, multiform-like erythema, erythema dyschromicum perstans-like, and erythema nodosum-like.^17^, ^18^

Sicca syndrome is the clinical manifestation of the autoimmune process against the salivary, lacrimal and eccrine glands. It occurs later in the disease, usually in older individuals. Fifty percent of the individuals with pSS have xerosis.^18^ There is reduced sweating with dry skin associated with symptoms such as pruritus, itching, and a burning sensation.^17^ There are some reports of cases of itching in the ear canal due to the reduced production of earwax and dermatitis due to eyelid dryness. Genital symptoms of Sicca syndrome include dyspareunia and vaginal pruritus.

Ocular and oral dryness occurs in 85% of patients with pSS.^18^ Xerostomia causes pain and a burning sensation in the oral mucosa, dysphagia, angular cheilitis, and a higher incidence of caries.

Raynaud’s phenomenon occurs in 30% of pSS cases and may be the first manifestation of the disease.

Cutaneous vasculitis occurs in 10% of pSS and in 85% of the cases it can be observed before the onset of Sicca syndrome symptoms.^18^ It is a small-vessel leukocytoclastic vasculitis and, when associated with cryoglobulinemia, it has a poor prognosis (more severe systemic disease, association with B lymphoma, and higher mortality rate). Clinically, it may present as typical purpuric lesions, but also as urticarial lesions of urticaria vasculitis, and rarely as erythema elevatum diutinum.^18^ Waldenström’s hypergammaglobulinemic purpura also occurs in the context of Sjögren’s syndrome and will be discussed further below in the purpura section.

Photosensitivity lesions, erythematous annular papules and plaques, and polycyclic annular erythema can occur in pSS (9% of cases) in individuals with anti-Ro antibodies (SSA) positivity, without association with LE. However, the differentiation (clinical and histopathological) of these lesions from subacute lupus lesions that have only skin disease is very difficult. Skin lesions are associated with a lower risk of glandular and systemic disease and have a better prognosis.^18^

The lesions may present an inflammatory infiltrate consisting of lymphocytes and plasma cells with or without reduction of eccrine glands and ductal structures, and perivascular lymphocytic infiltrate.^17^ Direct immunofluorescence shows epidermal deposits of intercellular IgG in 2/3 of pSS cases and in about 13% of sSS cases.^17^

Nodular localized cutaneous amyloidosis is a rare manifestation and in 25% of cases, it is associated with pSS. It is usually located on the lower and upper limbs, as well as on the trunk and face. It may accompany renal and lower respiratory tract amyloidosis.^18^

## Scleroderma

Scleroderma is a term that encompasses a series of diseases that manifest dermatologically with thickening of the skin. It can be part of a syndrome with vascular, pulmonary, cardiac, and renal manifestations (the systemic scleroses - SS) or be located only in the skin (the localized sclerodermas).

Systemic sclerosis is classified according to the latest ACR/EULAR (American College of Rheumatology/European League Against Rheumatism) consensus in 2013 as limited cutaneous systemic sclerosis and diffuse cutaneous systemic sclerosis.^19^ Their main characteristics are shown below:

Limited cutaneous SS: Raynaud’s phenomenon; skin induration on the distal extremities of the limbs, face (microstomy), and neck; telangiectasias; the presence of anti-centromere antibody and increased risk of pulmonary hypertension;

Diffuse cutaneous SS: recent onset of Raynaud’s Phenomenon; induration of the skin of the proximal portion of the limbs and trunk; early progression to cardiac involvement; pulmonary (fibrosis), renal and gastrointestinal involvement; tendon friction; increased risk of scleroderma renal crisis; the presence of Anti-Scl-70, an anti-topoisomerase antibody that increases the risk of pulmonary fibrosis; the presence of anti-RNA polymerase III antibody, which indicates a greater probability for renal crisis and cancer.

Histopathology of the affected skin is characteristic and demonstrates a reduction in the spaces between the collagen bundles, an increase in the thickness of the collagen bundles, without an increase in the number of fibroblasts. There is also entrapment of the eccrine glands by sclerosis, leading to the loss of their adipose pad. This change is preferentially located in the reticular dermis.

Non specific cutaneous manifestations related to systemic sclerosis are: capillaroscopy with SS pattern in 83%–98% of cases and with early onset; telangiectasias; calcinosis cutis; dyschromia: Addison’s disease-like hyperchromia, hypo- and hyperpigmented macules, and ‘salt and pepper’ leukoderma (which spares the perifollicular region).^20^

Localized scleroderma (also called morphea) comprises sclerosis of any depth in the skin and does not progress to systemic sclerosis. Twenty to 80% of individuals with morphea may have a positive ANA, and this is not associated with an increased risk of systemic disease or another connective tissue disease. There is no change in the periungual capillaries in localized scleroderma.

## Neutrophilic diseases

### Sweet’s Syndrome

Sweet’s syndrome is clinically characterized as erythematous lesions accompanied by fever, arthralgias/myalgias and leukocytosis, which can be recurrent.

**Figure 5:**
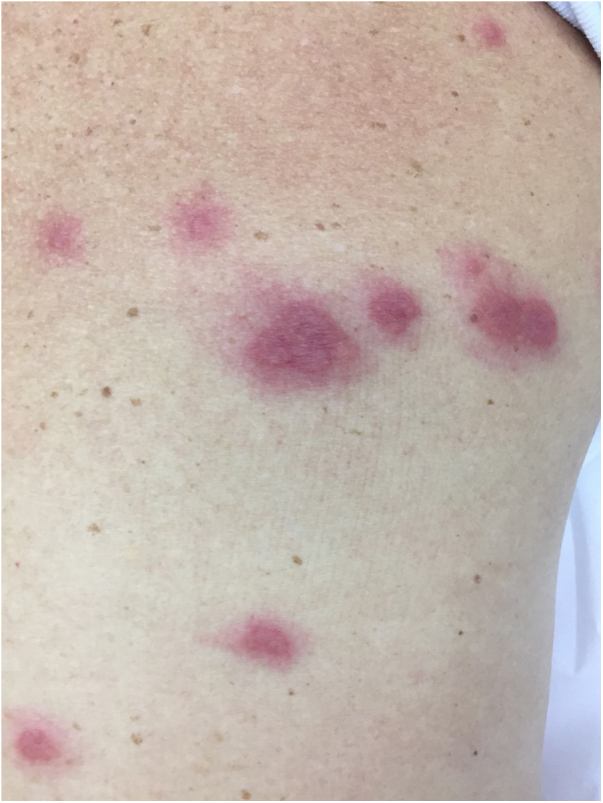
Sweet’s syndrome. Multiple erythematous papular infiltrated lesions with a pseudovesicular aspect in Sweet’s syndrome.

The characteristic (and histopathologically disease-specific) lesion is a well-defined erythematous plaque with a pseudovesicular, vesicular, or pustular surface (Fig. 5) Single lesions (especially in children) and deep nodular lesions can be less commonly observed. These nodules may have slight overlying erythema and are called subcutaneous Sweet’s syndrome. These nodules rarely ulcerate and, in these cases, they resemble the clinical appearance of pyoderma gangrenosum lesions. Their predominant distribution is on the face, trunk, and proximal extremities, but can occur in areas of trauma, characterizing a pathergy reaction.^21^ It is more frequent in women between 30 and 60 years of age.

In some cases, it is accompanied by a prodromal of flu-like symptoms. Extracutaneous symptoms may be observed, such as sterile osteomyelitis, conjunctivitis, ulcerative keratitis, and neutrophilic infiltration of the lungs simulating pneumonia, heart, nervous system, renal and gastrointestinal system.

It can be subdivided into classic, pararheumatic and paraneoplastic types and can also be related to pregnancy, medications and HIV infection. The hypothesis of paraneoplasia should be raised in the presence of any atypical manifestations, such as the presence of aphthous lesions in the oral mucosa, recurrence during corticoid weaning, high levels of ESR that do not reduce with corticotherapy, and in association with rheumatologic diseases.

Histopathological diagnostic criteria include the presence of diffuse neutrophilic infiltrate in the dermis, edema, and neutrophil fragmentation. Although the infiltrate may be more pronounced in perivascular areas, vasculitis is classically absent. The predominant cells in the dermal infiltrate are mature neutrophils, although eosinophils have been observed in some patients with classic or drug-induced Sweet’s syndrome. In histiocytoid Sweet’s syndrome, clinical lesions have a more annular configuration and, histopathologically, immature (myeloid) cells that resemble histiocytes are observed (it seems to be associated with hematological neoplasia).^21^

The main differential diagnoses are drug eruptions, erythema multiforme and leprosy (histopathology resembles a leprosy reaction).

### Behçet’s Disease

Behçet’s disease is a multisystemic vasculitis characterized by recurrent oral and genital ulcers, in addition to oral, joint, vascular, intestinal, and nervous system manifestations.^22^ It occurs in both genders and affects mainly young adults.

There are no pathognomonic laboratory tests or specific clinical findings. The diagnosis is made in the presence of the major criterion (recurrent oral aphthosis) associated with 2 of the 4 minor criteria (they are: recurrent genital ulcer; ocular lesions – uveitis or retinal vasculitis; skin lesions – erythema nodosum, pseudovasculitis, papulopustular lesions or acneiform nodules consistent with Behçet; and positive pathergy test).^23^

Posterior uveitis (i.e., retinal vasculitis) is the most often seen classic ocular form, although several other ocular findings can be observed. Arthritis seen in patients with Behcet’s disease is non-erosive and inflammatory and affects both large and small joints. The neurological manifestations are of late onset and have a remarkably variable presentation. Vasa vasorum vasculitis, with a tendency to affect large arteries and veins, can be a cause of death in these patients. Vessel thrombosis and aneurysms, likely due to chronic endovascular damage, are typically reported as a late disease manifestation. The kidneys are relatively spared in Behçet’s patients when compared to other types of vasculitis.

**Figure 6:**
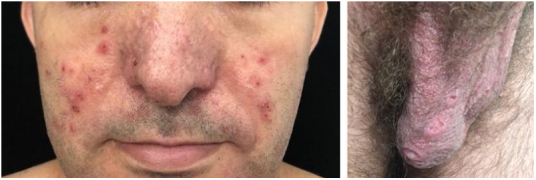
Behcet’s disease. Acneiform vesico-pustules on the face and ulcers on the scrotum (painful).

In the mucosa there are ulcerated aphthoid lesions. These are usually the first symptom of the disease (they may precede other symptoms by years) and also occur during disease activity. Genital ulcers occur on the penis, scrotum, and vulva. They are painful covered by fibrin and take a long time to heal (Fig. 6).

On the skin, the lesions comprise sterile vesicopustules or purpuric (non folliculocentric) acral and facial papules. Panniculitis lesions (erythema nodosum-like and histopathologically similar to nodular vasculitis) on the legs and gluteal region occur mainly in women. Erythema nodosum lesions themselves and thrombophlebitis lesions also occur in the context of Behcet’s disease. The phenomenon of pathergy is described, as well as in pyoderma gangrenosum.

Histopathology is non specific. The acneiform lesion may be a vasculopathy with thrombosis and neutrophilic vasculitis and intermixed histiocytic infiltrate. The oldest lesions show a predominance of lymphocytes. Subcutaneous nodular lesions comprise lobular panniculitis with or without septal involvement, adipocyte necrosis, in addition to the vascular findings described above.

### Bowel-associated dermatosis-arthritis syndrome (BADAS)

Bowel-associated dermatosis-arthritis syndrome (BADAS) is characterized by recurrent flare-ups of pustular lesions (follicular and non-follicular) and abscesses, synovitis, fever, and flu-like symptoms that develop after surgical procedures of the gastrointestinal tract (often bariatric surgery) or in patients with inflammatory bowel disease.^24^

The lesions are recurrent (every 4 to 6 weeks). Erythematous and purpuric macules, papules, and vesico-pustules may be seen on the proximal extremities and on the trunk. Also, painful and erythematous recurrent subcutaneous nodules (lobular neutrophilic panniculitis) are seen on the trunk and extremities, which heal with depressed atrophic scars. True erythema nodosum is also a manifestation of the disease.

On histopathology the initial lesions are not specific and the findings are very similar to Behçet’s disease lesions. There is papillary edema and subepidermal vesicles in more recent lesions, while older lesions show a dense neutrophilic dermal infiltrate.

### Pyoderma gangrenosum

**Figure 7:**
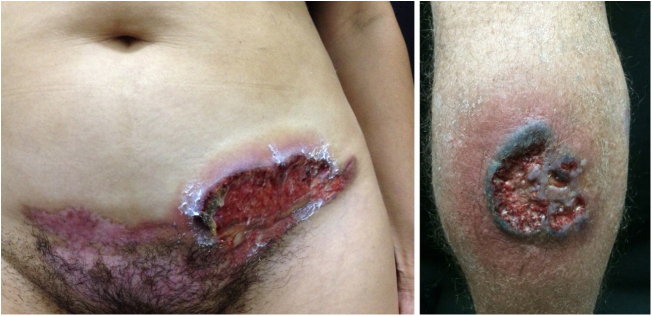
Pyoderma gangrenosum. Right: ulcerated lesion, with irregular, inflammatory and raised margins, dark red or purple in color and necrotic base (active lesion in pyoderma gangrenosum). Left: deep ulcer containing fibrin at the periphery, without a purplish edge (older lesion, in an area of C-section scar).

Pyoderma gangrenosum is a rare neutrophilic dermatosis characterized by rapidly progressive skin ulcers. These ulcers expand peripherally, are painful, and have well-defined edges of erythematous to violaceous color (Fig. 7). The pathergy reaction can be observed and, when it regresses, the atrophic scar acquires a cribriform pattern. As histopathology is non specific, the diagnosis is one of exclusion. Table 3 describes the systemic conditions associated with pyoderma gangrenosum.^25^

**Table 3:** Diseases associated with pyoderma gangrenosum.

**Hematological**	Myelofibrosis
	Myelocytic leukemia
	Agnogenic myeloid metaplasia
	Hairy cell leukemia
	IgA monoclonal gammopathy
	Polycythemia vera
	Multiple myeloma
**Rheumatological**	Seronegative arthritis with inflammatory bowel disease
	Seronegative arthritis without inflammatory bowel disease
	Rheumatoid arthritis
	Osteoarthritis
	Spondyloarthropathies
	Systemic lupus erythematosus
	Takayasu’s arteritis
**Gastrointestinal tract**	Ulcerative colitis
	Crohn’s disease
	Chronic active hepatitis
	Regional enteritis
	Primary biliary cirrhosis
**Endocrinological**	Diabetes mellitus
	Thyroid diseases
**Others**	Auto-inflammatory syndromes with:
	Suppurative hidradenitis;
	Neutrophilic dermatoses;
	Acne conglobate
	Paroxysmal nocturnal hemoglobinuria
	Sarcoidosis
	Neoplasms
	Lung diseases

Any disease that presents with ulcers are included in the differential diagnosis of pyoderma gangrenosum, such as leishmaniasis and sporotrichosis. In cases clinically similar to pyoderma gangrenosum, but which behave differently, one should consider the possibility of granulomatosis with polyangiitis. Furthermore, in the case of pyoderma gangrenosum refractory to the traditional therapy, an auto-inflammatory disease should be considered.

Pyoderma gangrenosum has some variants such as:

- Pyostomatitis vegetans: chronic, pustular and eventually vegetating erosions on the mucous membranes;
- Atypical or bullous pyoderma gangrenosum: more superficial ulcerations on the upper extremities and face. Described in hematologic disorders such as myelodysplastic syndrome or acute myeloid leukemia;
- Periostomal pyoderma gangrenosum.

### Erythema elevatum diutinum

It is a rare cutaneous leukocytoclastic vasculitis that initially presents as erythematous-violet papules that coalesce to form yellowish erythematous plaques resembling urticarial lesions. The lesions tend to appear in areas of trauma and to respect a symmetrical distribution on the extensor surfaces of the extremity joints and the gluteal region. The condition may be associated with arthralgias and arthritis.

Hematological malignancies are considered the most commonly associated factors, the most frequent of which is monoclonal IgA gammopathy.^26^

It has also been reported in association with neoplastic, autoimmune, and infectious diseases, especially acquired immunodeficiency virus infection. In the latter, the clinical presentation may be altered with the presence of nodular lesions and palmoplantar involvement.^27^

## Purpuras

Purpuras can be associated with both severe and life-threatening diseases and common, benign chronic conditions. In both cases, they are characterized as non specific manifestations of a myriad of diseases. Anyway, in most cases, a detailed anamnesis and clinical examination associated with some laboratory tests are enough for its evaluation.

The morphology of purpura helps both in its differentiation and in understanding its pathophysiological mechanism. The purpuras may be the result of a simple hemorrhagic process, hemorrhage secondary to vessel inflammation and, finally, occlusive hemorrhage with minimal secondary inflammation.

### Simple petechial hemorrhage

Thrombocytopenia or platelet dysfunction: ecchymoses may be observed, but the predominant morphology is petechia. Petechiae may appear with platelet counts <50,000 mm^3^; however, they are typically seen with platelet counts ≤10,000 mm^3^. Severe thrombocytopenia results in the deterioration of the endothelial adhesion in vessels leading to an increase in vascular permeability with extravasation of red blood cells.

Platelet function disorders, on the other hand, more often lead to ecchymoses secondary to minimal trauma than to the appearance of petechiae.^28^

### Intravascular pressure surges

Severe or repetitive surges in intravascular pressure can result in petechial hemorrhage such as that seen above the claviculae after labor, coughing or vomiting fits, and excessive crying in children. The use of tourniquets can lead to the local distribution of petechiae (above the compressed place).

### Minimally inflammatory microvascular syndromes

Chronic pigmentary purpura and Waldenström’s hypergammaglobulinemic purpura affect smaller-caliber vessels in the dermis. In the first one, an area of ​​recurrent hemorrhage is observed, often petechial with adjacent erythema and brownish hyperpigmentation as a result of hemosiderin deposition, which results in a residual ocher discoloration. Depending on the clinical aspect of the underlying inflammatory process, it can be called Schamberg’s purpura – with punctiform hemosiderin resulting in a “cayenne pepper” appearance; Doucas-Kapetanakis telangiectatic purpura – purpuric desquamative macules or papules; Purpura annularis telangiectodes of Majocchi – purpuric lesions of annular shape; lichen aureus – single purpuric to brown plaque or, if older, ocher; and, more rarely, the variants: linear pigmented purpura and granulomatous pigmentary purpura.

Waldenström’s hypergammaglobulinemic purpura is characterized by macular hemorrhage in gravity-dependent areas and areas covered by tight clothing, which may be accompanied by a local burning sensation. It can be idiopathic or associated with conditions that course with polyclonal gammopathies, such as Sjögren’s syndrome, sarcoidosis, and other conditions.^29^

### Problems in the coagulation cascade

#### Vascular causes

Inflammatory causes: in these cases, inflammation affecting the vessels is observed. Perivascular inflammation is not considered vasculitis and does not result in palpable purpura. If lesions are secondary to immune complex deposits, they can commonly be seen in gravity-dependent areas. An important cause of palpable purpura is leukocytoclastic vasculitis, which can be observed in several conditions (discussed below).

Non-inflammatory causes: painful retiform purpura (arboriform, stellate) with acute presentation is typical of antiphospholipid antibody syndrome and other thrombotic vasculopathies. They usually manifest on the extremities, auricles, cheeks, forehead, and trunk, or they can be disseminated. Occasionally, some lesions may show peripheral erythema and necrotic bullae. Histopathology shows non inflammatory diffuse thrombosis of the dermal vessels, with no evidence of vasculitis.^30^

#### Extravascular causes

Major trauma can result in cutaneous bleeding. In these cases, edema, pain, and abrasions suggest the diagnosis. Purpuras related to minimal trauma usually occur as a result of defective connective tissue that offers little support to the small vessels of the skin, as seen in actinic purpura, due to excess of corticosteroids (local or systemic), in Ehlers-Danlos syndrome, in elastic pseudoxanthoma, and in light-chain amyloidosis. In the latter, purpura is easily induced by pinching the skin.

The presence of perifollicular hemorrhage suggests the diagnosis of scorbutus.

## Vasculitis

Vasculitis is a consequence of blood vessel inflammation.^31^ It may indicate a disease limited to the skin, the manifestation of a systemic disease or drug use, or a primary skin disease with systemic effects. Vasculitis can be caused by infections, drugs, neoplasms, autoimmune and connective tissue diseases. In these cases, it constitutes a non specific reaction to the causal agent. However, approximately half of the cases are idiopathic leukocytoclastic vasculitis.

The clinical effects depend on the location and size of the affected vessel.^32^

The 2012 International Chapel Hill Consensus Conference on the Nomenclature of Systemic Vasculitides defined the nomenclature of systemic vasculitis.^32^ They are classified as:

### Primary vasculitis

Small-vessel vasculitis: related to ANCA (microscopic polyangiitis, granulomatosis with polyangiitis, eosinophilic granulomatosis with polyangiitis) and associated with immune complex deposits (IgA vasculitis, cryoglobulinemic vasculitis, hypocomplementemic urticarial vasculitis, anti-glomerular basement membrane disease).

Medium-vessel vasculitis: polyarteritis nodosa and Kawasaki disease.

Large-vessel vasculitis: Takayasu’s arteritis.

Vasculitis of variable size vessels: Behçet’s disease and Cogan’s syndrome.

Single-organ vasculitis: leukocytoclastic vasculitis limited to the skin, cutaneous arteritis, isolated aortitis, primary central nervous system vasculitis.

Cutaneous vasculitis can manifest with changes in skin color, such as erythema, petechiae and livedo reticularis, and even cell damage in all layers, such as in digital gangrene.

When the small vessels usually located in the papillary dermis are involved, they cause a maculopapular exanthema that progresses to palpable purpura. However, these lesions do not disappear with pressure. Macules, petechiae, vesicles, bullae, and ulcerations have also been described.^31^

Damage to the medium sized vessels located in the deep dermis causes greater and deeper damage. Nodules, ulcers, livedo, and necrotic lesions have also been described. There are no large vessels in the skin.^31^

The most common clinical presentation of cutaneous vasculitis is palpable purpura and the distal region of the lower limbs is the most predominant area. The main extracutaneous manifestations are arthralgias and arthritis, renal alterations and gastrointestinal involvement.

#### Takayasu’s arteritis

Takayasu’s arteritis is a large-vessel vasculitis, but it can affect smaller vessels. Inflammation progresses to occlusion that can lead to stenosis or aneurysm formation. It is more common in women and usually starts in the second and third decades of life. On the skin, it can cause purpura, livedo reticularis, subcutaneous lesions similar to erythema nodosum, indurated erythema, inflammatory nodules, necrotic and/or ulcerated nodules, digital gangrene, ulcers similar to pyoderma gangrenosum, Raynaud’s phenomenon, urticaria, angioedema, and erythema multiforme.^32^, ^33^ The nodules are usually unrelated to the vascular involvement site and can arise at any stage of the disease. Cases of association with Sweet's syndrome have been described in children.^33^

#### Giant cell arteritis

The cutaneous manifestations of giant cell arteritis are rare. They may be ischemic lesions resulting from arterial obstruction or lesions generated by other mechanisms. Induration, erythema and bullae on the scalp and temples, glossitis and tongue necrosis, scalp necrosis, purpura, distal limb gangrene, periorbital ecchymosis, face and neck edema, nodules, and granuloma annulare have been described.^33^

#### Polyarteritis nodosa

Systemic polyarteritis nodosa (PAN) is a necrotizing vasculitis of small or medium-sized arteries without glomerulonephritis or vasculitis of arterioles, capillaries or venules, not associated with ANCA. Palpable purpura, livedo reticularis, nodules, urticarial lesions, transient erythema, superficial phlebitis, distal necrosis, and splinter hemorrhages can be observed.^33^

Cutaneous PAN is a necrotizing vasculitis of deep dermal and subcutaneous arterioles. It may be related to infections (hepatitis B, hepatitis C, *M. tuberculosis*, HIV, HTLV, *parvovirus* B19, cytomegalovirus; and in children: streptococcosis), inflammatory diseases, rheumatoid arthritis, and drugs (penicillin, minocycline). The main findings are subcutaneous nodules, livedo reticularis, purpura, and ulcers.^33^ Symptoms such as fever, weight loss, arthritis, and neuropathy can appear in any presentation.

#### Kawasaki disease

Kawasaki disease is an acute vasculitis that occurs mainly in children. It usually starts with fever and a polymorphic exanthema of centrifugal dissemination, in addition to oropharyngeal enanthema and ‘strawberry tongue’. After a few days, perineal and fingertip desquamation appears.^33^ The disease can progress to vasculitis and aneurysm formation in the coronary arteries. Therefore, diagnostic suspicion is essential for the early implementation of treatment and minimization of sequelae.

#### ANCA-associated vasculitis

ANCA-associated vasculitis most commonly causes palpable purpura, but may occasionally present with livedo racemosa, erythema nodosum, subcutaneous nodules, and painful ulcers. Neutrophil-predominant leukocytoclastic vasculitis is the most common histopathological finding in microscopic polyangiitis and in granulomatosis with polyangiitis (GPA) – formerly called Wegener’s granulomatosis. In eosinophilic granulomatosis with polyangiitis (formerly called Churg-Strauss syndrome), extravascular necrotizing granulomas with mixed neutrophil and eosinophil infiltrates predominate.^32^, ^33^

#### Vasculitis associated with immune complexes

IgA vasculitis, better known in children as Henoch-Schönlein purpura, is the most common vasculitis in childhood and usually presents as erythematous papules that take an ascending direction from the distal region of the lower limbs and progress to palpable purpura.

**Figure 8:**
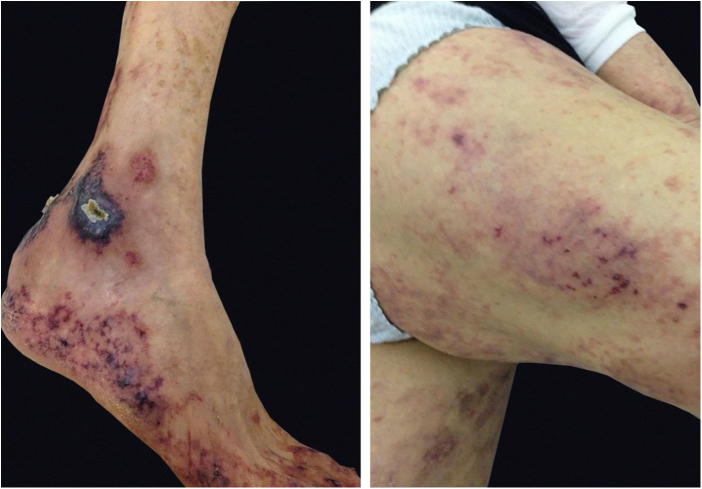
Cryoglobulinemic thrombotic vasculopathy. Type 1 cryoglobulinemia coursing with thrombotic vasculopathy: erythematous and purpuric macules with jagged outline and central necrosis.

Cryoglobulinemic vasculitis is caused by the deposition of cryoglobulins in small vessels. According to the deposited cryoglobulins, they can be divided into type I (monoclonal), type II (mono and polyclonal), or type III (polyclonal).^32^ Type I is related to lymphoproliferative disorders of B lymphocytes, such as multiple myeloma, and manifests as a thrombotic vasculopathy (Fig. 8). Types II and III are associated with infections (mainly hepatitis C), autoimmunity (rheumatoid arthritis, Sjögren’s syndrome, and systemic lupus erythematosus), or neoplastic diseases, but they may also be idiopathic.^34^ The idiopathic type III is called essential mixed cryoglobulinemia. The most common clinical form of cryoglobulinemia is the intermittent appearance of palpable petechiae in the lower limbs. Acrocyanosis, necrosis, and painful ulcers may also appear,^32^ with or without arthralgia, arthritis, neuropathy, and nephropathy.

Hypocomplementemic urticarial vasculitis presents with urticarial plaques that last for more than 24 hours, are often more painful than pruriginous, and leave local pigmentation. It is more common in women and may be accompanied by arthralgia and ocular changes. For diagnosis, recurrent urticarial lesions and reduced serum complement must be present, as well as two of the following characteristics: cutaneous vasculitis (leukocytoclastic), arthritis or arthralgias, ocular inflammation, glomerulonephritis, abdominal pain, and/or anti-C1q antibody. Hepatitis B, neoplasms, systemic lupus erythematosus, and Sjögren’s syndrome should be investigated.

#### Variable vessel vasculitis

Behcet’s disease is a disorder associated with immune-mediated obstructive vasculitis that was addressed in the section on neutrophilic diseases.

Cogan Syndrome is an ocular and auditory immune disease that may be associated with systemic vasculitis.

### Secondary vasculitis

They are associated with systemic diseases (SLE, rheumatoid arthritis, sarcoidosis).

Vasculitis associated with a probable etiology: neoplasms, infections, drug-related (associated with ANCA or immune complex deposits).

Table 4 describes the types of vasculitis that occur in the context of some systemic diseases.^31^, ^32^, ^35^, ^36^, ^37^, ^38^, ^39^, ^40^, ^41^, ^42^

**Table 4:** Secondary vasculitis.

**Lupus erythematosus**	Cutaneous leukocytoclastic vasculitis: erythematous macules and papules, palpable purpura, ischemic or ulcerated lesions, urticarial lesions and nodules.^31^, ^32^
**Rheumatoid arthritis**	Cutaneous leukocytoclastic vasculitis: palpable purpura, ulcers, ischemic digital lesions, maculopapular erythema, hemorrhagic bullae, erythema elevatum diutinum, livedo reticularis, subcutaneous nodules, and livedoid vasculitis.^31^, ^32^
**Sarcoidosis**	Leukocytoclastic vasculitis with or without granulomas, livedo reticularis, lower-limb ulcers and purpuric or annular lesions.^35^, ^36^
**Sjögren syndrome**	Leukocytoclastic vasculitis, urticarial vasculitis and cryoglobulinemic vasculitis.^31^, ^37^
**Erythema elevatum diutinum**	Chronic vasculitis.
**Medications (resolution with drug withdrawal)**	anti-TNFa, levamisole, propylthiouracil, hydralazine, rituximab, montelukast, statins.^39^
**Neoplasms**	Paraneoplastic syndromes (lymphoproliferative and myeloproliferative diseases)
**Others**	Dermatomyositis
	Systemic sclerosis
	Inflammatory bowel diseases
	COVID (small-vessel cutaneous leukocytoclastic vasculitis, urticarial vasculitis and perniosis-like lymphocytic vasculitis)^40^

## Granulomatous diseases

### Sarcoidosis

It has an unknown cause and pathogenesis. It is believed that some environmental agent (virus, bacteria, autoantigens, silica, pollen) has an effect on the genetically-susceptible host, activating the immune response, resulting in granuloma formation. It is present on the skin in 2 5% to 30% of cases, and of these, it affects some other organs in 50%.^43^

Sarcoidosis-specific lesions are the ones that, upon being biopsied, reveal non caseating epithelioid granulomas and are characterized by small, erythematous-violaceous papules (most common presentation, located on the head and neck, periorbital, perinasal, and perioral areas) similar to those of discoid CCLE, plaques, nodules, subcutaneous nodules (Darier-Roussy disease: asymptomatic, on the trunk and extremities, with or without a violaceous epidermis), lesions over scars, papules associated with tattoos, and lupus pernio (lesions infiltrating the nose and chin). When the involvement is extensive, there is a higher rate of respiratory disease and bone cysts. There may also be damage to the nasal mucosa and underlying bone. This form is the most refractory to treatment. Other specific lesions, albeit rarer, are: acquired ichthyosis, erythroderma, psoriasiform lesions, ulcers, verrucous lesions, alopecia, photo distributed lesions, and angiolupoid sarcoidosis (lupus pernio lesions with telangiectasias; greater chance of ulceration and association with ocular disease). Sarcoidosis is known as the “great imitator”. The non specific lesion is represented by erythema nodosum, with symmetrical involvement, in the pretibial region, with a good prognosis. It may be accompanied by uveitis, fever, arthritis, and bilateral hilar lymphadenomegaly, characterizing Löfgren’s syndrome (an acute form that resolves spontaneously in up to 90% of cases and which, due to its specificity, does not require a biopsy to establish the diagnosis). Other associated syndromes are: Heerfordt-Waldenström (fever, parotid enlargement, anterior uveitis, and facial nerve palsy), Mikulicz (involvement of the lacrimal, sublingual, submandibular, and parotid glands) and sarcoidosis-lymphoma (development of a Hodgkin or non-Hodgkin lymphoma in a patient with chronic sarcoidosis; it is crucial to differentiate it from sarcoidosis-like lesions triggered by monoclonal antibodies used against neoplasms).^44^

All cases of cutaneous sarcoidosis should be investigated for systemic disease. The diagnosis is made by combining the clinical picture, radiological and laboratory findings, diascopy (‘apple jelly’ appearance), and biopsy (histopathology with non caseating granulomas), with the skin lesion providing the easiest access for histopathological assessment. However, the biopsy of palpable lymph nodes and salivary gland is also indicated to assess systemic involvement.

The mortality rate is around 3% to 6%, due to cardiac, pulmonary, and neurological involvement.

### Necrobiosis lipoidica

This is a chronic inflammatory granulomatous disease, associated with connective tissue degeneration, commonly seen in association with diabetes, hypertension, thyroid disease, and obesity. It is three times more frequent in women.^44^

The clinical picture presents as irregular, ovoid plaques, with a violaceous, hardened periphery and an atrophic, yellow, and shiny center. Surface telangiectasias are common. It may be associated with hypohidrosis, alopecia, and anesthesia. It is more frequent in the pretibial region, and ulceration is common, even with minimal trauma.

The diagnosis is attained with clinical examination and biopsy of the lesion edge, including the subcutaneous tissue, which detects interstitial palisaded granulomas, consisting of histiocytes and multinucleated giant cells in the dermis and subcutaneous tissue, associated with collagen degeneration.

### Granuloma annulare

Non infectious granulomatous skin disease, associated with several diseases, such as diabetes, thyroid disease, dyslipidemia, infections (HIV and borreliosis), and malignancies, resulting in the need for further investigation. It seems to be a cell-mediated reaction to an unknown antigen, with possible triggers, including trauma, drugs (including cancer immunotherapy), vaccination, and ultraviolet light.^44^ The clinical picture can be localized (75% of cases, with skin-colored, erythematous-violaceous or brownish, non desquamative papules that coalesce to form annular plaques), generalized (affecting the trunk and extremities, with greater refractoriness, later onset, and which may include a burning sensation) and a subcutaneous form (more on lower limbs of children, with a differential diagnosis with rheumatoid nodule).^45^ The most atypical forms are: perforating granuloma annulare (ulcerated, umbilicated, or with a crusted/central scale), palmoplantar, pustular, visceral.

It is usually asymptomatic and treatment is mostly for cosmetic reasons.^46^ The investigation must be carried out with laboratory tests, including whole blood count, thyroid hormones, glycosylated hemoglobin, lipid profile, and serology for hepatitis B, C, and HIV. Screening for cancer should be carried out according to the patient’s age.

## Financial support

None declared.

## Authors’ contributions

Ana Luisa Sampaio: Approval of the final version of the manuscript; drafting and editing of the manuscript; collection, analysis, and interpretation of data; critical review of the literature; critical review of the manuscript.

Aline Lopes Bressan: Approval of the final version of the manuscript; drafting and editing of the manuscript; collection, analysis, and interpretation of data; critical review of the literature.

Barbara Nader Vasconcelos: Approval of the final version of the manuscript; drafting and editing of the manuscript; collection, analysis, and interpretation of data; critical review of the literature.

Alexandre Carlos Gripp: Approval of the final version of the manuscript; design and planning of the study; drafting and editing of the manuscript; collection, analysis, and interpretation of data; critical review of the literature; effective participation in research orientation.

## Conflicts of interest

None declared.
